# Supplementary material for: Inconsistent year-to-year fluctuations limit the conclusiveness of global higher education rankings for university management
Source: PeerJ. 2015 Aug 25;3:e1217. doi: 10.7717/peerj.1217 (PMC4556149; doi:10.7717/peerj.1217)
Supplement: Supplemental Information 1 [file peerj-03-1217-s001.docx]

**Supplementary information**

**Table S1 a-h) Regression Models THES Ranking**

| Table S1 a) (plot figure 1a) |  |  |  |  |  |
| --- | --- | --- | --- | --- | --- |
|  | **Estimate** | **2.5% \| 97.5%** | **Std. Error** | **t value** | **P** |
| (Intercept) | -6.75246 |  | 1.93857 | -3.483 | 0.000628 |
| Score2010 | 1.06831 | 1.008 \| 1.13 | 0.03074 | 34.754 | P < 0.0001 |
| **R-squared:** | 0.8746 |  |  |  |  |
| 172 degrees of freedom |  |  |  |  |  |
|  |  |  |  |  |  |
| Table S1 b) (plot figure 1b) |  |  |  |  |  |
|  | **Estimate** | **2.5% \| 97.5%** | **Std. Error** | **t value** | **P** |
| (Intercept) | 9.51167 |  | 0.92447 | 10.29 | P < 0.0001 |
| Score 2011 | 0.89906 | 0.087 \| 0.929 | 0.01534 | 58.61 | P < 0.0001 |
| **R-squared:** | 0.9478 |  |  |  |  |
| 188 degrees of freedom |  |  |  |  |  |
|  |  |  |  |  |  |
| Table S1 c) (plot figure 1c) |  |  |  |  |  |
|  | **Estimate** | **2.5% \| 97.5%** | **Std. Error** | **t value** | **P** |
| (Intercept) | -2.34962 |  | 0.89422 | -2.628 | 0.00931 |
| Score2012 | 0.97488 | 0.95 \| 1.003 | 0.01408 | 69.258 | P < 0.0001 |
| **R-squared:** | 0.9619 |  |  |  |  |
| 189 degrees of freedom |  |  |  |  |  |
|  |  |  |  |  |  |
| Table S1 d) (plot figure 1d) |  |  |  |  |  |
|  | **Estimate** | **2.5% \| 97.5%** | **Std. Error** | **t value** | **P** |
| (Intercept) | 3.26961 |  | 0.56031 | 5.835 | P < 0.0001 |
| Score2013 | 0.961868 | 0.94 \| 0.98 | 0.009345 | 102.923 | P < 0.0001 |
| **R-squared:** | 0.9827 |  |  |  |  |
| 185 degrees of freedom |  |  |  |  |  |
|  |  |  |  |  |  |
| Table S1 e) (plot figure 1e) |  |  |  |  |  |
|  | **Estimate** | **2.5% \| 97.5%** | **Std. Error** | **t value** | **P** |
| (Intercept) | 14.25097 |  | 4.398 | 3.24 | 0.00143 |
| Rank2010 | 0.85529 | 0.77 \| 0.93 | 0.04115 | 20.79 | P < 0.0001 |
| **Adjusted R-squared:** | 0.7136 |  |  |  |  |
| 172 degrees of freedom |  |  |  |  |  |
|  |  |  |  |  |  |
| Table S1 f) (plot figure 1f) |  |  |  |  |  |
|  | **Estimate** | **2.5% \| 97.5%** | **Std. Error** | **t value** | **P** |
| (Intercept) | 7.11189 |  | 3.11633 | 2.282 | 0.0236 |
| Rank2011 | 0.93003 | 0.875 \| 0.99 | 0.02809 | 33.107 | P < 0.0001 |
| **R-squared:** | 0.8528 |  |  |  |  |
| 189 degrees of freedom |  |  |  |  |  |
|  |  |  |  |  |  |
| Table S1 g) (plot figure 1g) |  |  |  |  |  |
|  | **Estimate** | **2.5% \| 97.5%** | **Std. Error** | **t value** | **P** |
| (Intercept) | 3.35139 |  | 2.18742 | 1.532 | 0.127 |
| Rank2012 | 0.96595 | 0.93 \| 1.005 | 0.01969 | 49.049 | P < 0.0001 |
| **R-squared:** | 0.9268 |  |  |  |  |
| 185 degrees of freedom |  |  |  |  |  |
|  |  |  |  |  |  |
|  |  |  |  |  |  |
| Table S1 h) (plot figure 1h) |  |  |  |  |  |
|  | **Estimate** | **2.5% \| 97.5%** | **Std. Error** | **t value** | **P** |
| (Intercept) | 1.36263 |  | 1.91355 | 0.712 | 0.477 |
| Rank2013 | 1.00949 | 0.974 \| 1.044 | 0.01755 | 57.507 | P < 0.0001 |
| **R-squared:** | 0.9467 |  |  |  |  |
| 172 degrees of freedom |  |  |  |  |  |

**Table S2 a-h) Regression Models ARWU Ranking**

| Table S2 a) (plot figure 3a) |  |  |  |  |  |
| --- | --- | --- | --- | --- | --- |
|  |  |  |  |  |  |
|  | **Estimate** | **2.5% \| 97.5%** | **Std. Error** | **t value** | **P** |
| (Intercept) | 0.058263 |  | 0.216634 | 0.269 | 0.789 |
| score2010 | 1.000911 | 0.99\|1.012 | 0.005528 | 181.075 | P < 0.0001 |
| **AdjustedR-squared:** | 0.9971 |  |  |  |  |
| 95 degrees of freedom |  |  |  |  |  |
|  |  |  |  |  |  |
|  |  |  |  |  |  |
| Table S2 b) (plot figure 3b) |  |  |  |  |  |
|  |  |  |  |  |  |
|  |  |  |  |  |  |
|  | **Estimate** | **2.5% \| 97.5%** | **Std. Error** | **t value** | **P** |
| (Intercept) | -0.214665 |  | 0.172703 | -1.243 | 0.217 |
| score2011 | 1.000339 | 0.99 \| 1.009 | 0.004375 | 228.671 | P < 0.0001 |
| **AdjustedR-squared:** | 0.9982 |  |  |  |  |
| 94 degrees of freedom |  |  |  |  |  |
|  |  |  |  |  |  |
|  |  |  |  |  |  |
|  |  |  |  |  |  |
| Table S2 c) (plot figure 3c) |  |  |  |  |  |
|  |  |  |  |  |  |
|  | **Estimate** | **2.5% \| 97.5%** | **Std. Error** | **t value** | **P** |
| (Intercept) | 0.20383 |  | 0.166838 | 1.222 | 0.225 |
| score2012 | 0.992015 | 0.98 \| 1.000 | 0.004271 | 232.272 | P < 0.0001 |
| **AdjustedR-squared:** | 0.9982 |  |  |  |  |
| 96 degrees of freedom |  |  |  |  |  |
|  |  |  |  |  |  |
|  |  |  |  |  |  |
|  |  |  |  |  |  |
| Table S2 d) (plot figure 3d) |  |  |  |  |  |
|  |  |  |  |  |  |
|  | **Estimate** | **2.5% \| 97.5%** | **Std. Error** | **t value** | **P** |
| (Intercept) | 0.47687 |  | 0.333487 | 1.43 | 0.156 |
| score2013 | 0.984078 | 0.97 \| 1.000 | 0.008458 | 116.35 | P < 0.0001 |
| **AdjustedR-squared:** | 0.9937 |  |  |  |  |
| 85 degrees of freedom |  |  |  |  |  |
|  |  |  |  |  |  |
|  |  |  |  |  |  |
|  |  |  |  |  |  |
| Table S2 e) (plot figure 3e) |  |  |  |  |  |
|  |  |  |  |  |  |
|  | **Estimate** | **2.5% \| 97.5%** | **Std. Error** | **t value** | **P** |
| (Intercept) | 0.1128 |  | 0.87078 | 0.13 | 0.897 |
| rank2010 | 1.00441 | 0.97 \| 1.034 | 0.01531 | 65.59 | P < 0.0001 |
| **R-squared:** | 0.9782 |  |  |  |  |
| 95 degrees of freedom |  |  |  |  |  |
|  |  |  |  |  |  |
|  |  |  |  |  |  |
|  |  |  |  |  |  |
| Table S2 f) (plot figure 3f) |  |  |  |  |  |
|  |  |  |  |  |  |
|  | **Estimate** | **2.5% \| 97.5%** | **Std. Error** | **t value** | **P** |
| (Intercept) | 0.190909 |  | 0.553378 | 0.345 | 0.731 |
| rank2011 | 0.997138 | 0.98 \| 1.016 | 0.009892 | 100.798 | P < 0.0001 |
| **R-squared:** | 0.9907 |  |  |  |  |
| 94 degrees of freedom |  |  |  |  |  |
|  |  |  |  |  |  |
|  |  |  |  |  |  |
| Table S2 g) (plot figure 3g) |  |  |  |  |  |
|  |  |  |  |  |  |
|  | **Estimate** | **2.5% \| 97.5%** | **Std. Error** | **t value** | **P** |
| (Intercept) | 0.314811 |  | 0.475385 | 0.662 | 0.509 |
| rank2012 | 0.991143 | 0.97 \| 1.007 | 0.008358 | 118.585 | P < 0.0001 |
| **R-squared:** | 0.9931 |  |  |  |  |
| 96 degrees of freedom |  |  |  |  |  |
|  |  |  |  |  |  |
|  |  |  |  |  |  |
|  |  |  |  |  |  |
| Table S2 h) (plot figure 3h) |  |  |  |  |  |
|  |  |  |  |  |  |
|  | **Estimate** | **2.5% \| 97.5%** | **Std. Error** | **t value** | **P** |
| (Intercept) | 1.28349 |  | 1.06017 | 1.211 | 0.229 |
| rank2013 | 0.96857 | 0.929 \| 1.007 | 0.01953 | 49.586 | P < 0.0001 |
| **R-squared:** | 0.9662 |  |  |  |  |
| 85 degrees of freedom |  |  |  |  |  |

Table S3) (plot figure 7).

|  | **Estimate** | **2.5% \| 97.5%** | **Std. Error** | **t value** | **P** |
| --- | --- | --- | --- | --- | --- |
| (Intercept) | 12.3458 |  | 4.2609 | 2.897 | 0.00506 |
| THES | 0.7265 | 0.56 \| 0.89 | 0.0841 | 8.638 | 1.52E-12 |
| R-squared | 0.5232 |  |  |  |  |


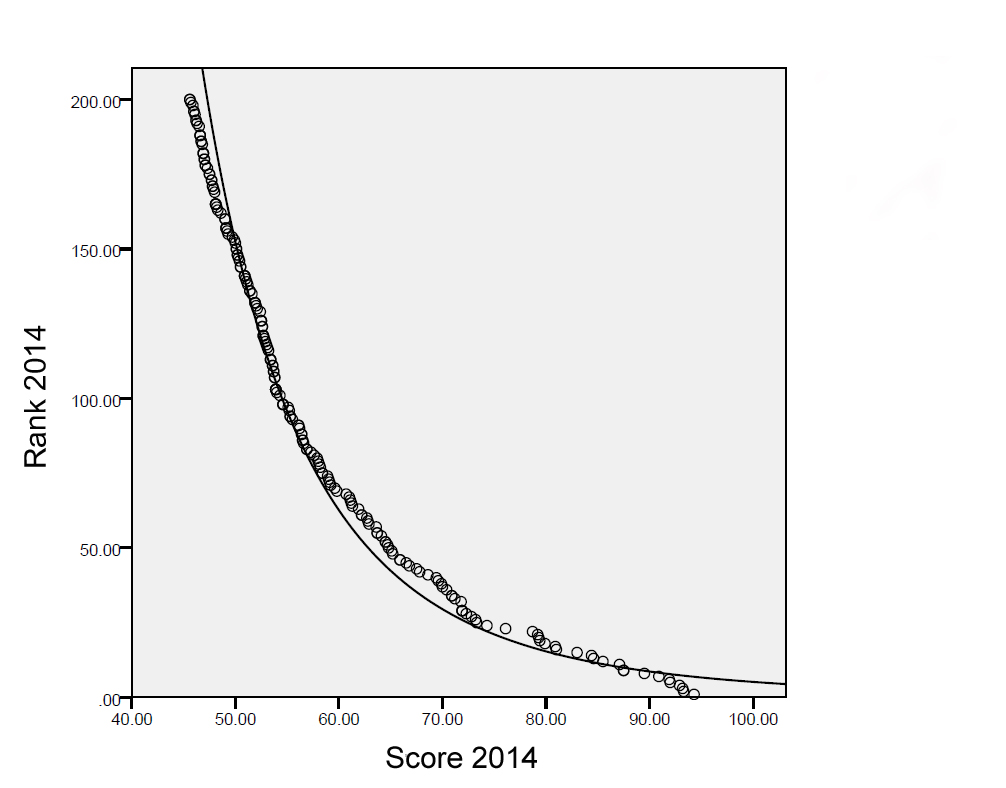


Figure S1) Scores vs ranks including power fit for the ranking 2014.

Table S3)

The 20 universities that are showing the total highest fluctuation in the THES rankings, from 2010 to 2014 (sum of changed ranks from 2010 to 2014).

| University | Sum of Rank Changes from 2010 to 2014 |
| --- | --- |
| Technical University of Denmark | 121 |
| Albert-Ludwigs-Universität Freiburg | 121 |
| Yeshiva University | 118 |
| University of Massachusetts | 117 |
| Universität Basel | 116 |
| Stony Brook University | 110 |
| University of Groningen | 109 |
| University of Glasgow | 108 |
| University of Zürich | 107 |
| National Tsing Hua University | 107 |
| George Washington University | 105 |
| University of Virginia | 104 |
| Boston College | 103 |
| Seoul National University | 101 |
| Uppsala University | 97 |
| University of California, Santa Cruz | 95 |
| Arizona State University | 93 |
| Pennsylvania State University | 89 |
| Erasmus University Rotterdam | 89 |
| Georgetown University | 89 |

Table S4)

The 10 universities that are showing the total highest fluctuation in the ARWU rankings, from 2010 to 2014 (sum of changed ranks from 2010 to 2014).

| University | Sum of Rank Changes from 2010 to 2014 |
| --- | --- |
| Hebrew University Jerusalem | 36 |
| Australische National University | 27 |
| University Aarhus | 24 |
| Technische Universität München | 21 |
| Ludwig-Maximilians-Universität München | 21 |
| University Uppsala | 20 |
| Universityt Gent | 20 |
| Rice University | 19 |
| Carnegie Mellon University | 18 |
